# Society Meetings

**Published:** 1896-02

**Authors:** 


### Society Meetings.

The Association of Medical officers of the National Guard and Naval Militia held its third annual meeting at the armory of the Tenth Battalion, at Albany, January 15, 1896. Surgeon-General Terry presided and among the medical officers present were: Colonel N. H. Henry, Majors W. E. Lambert, B. S. Beach, Herman

Bendell, A. H. Briggs, F. L. R. Tettaman, W. E. Spencer, Lewis Balch; Captains A. R. Jarrett, C. D. Napier; Lieutenants F. L. Fuchs, H. C. Baun, E. Colton, D. S. Burr, M. C. Ashley, D. C. Dye and R. W. Warner. The various suggestions of the surgeon-general, based on the reports of the committees appointed by him, were debated and measures were adopted looking to the elevation of the medical arm of the guard.

